# Deep-Learning-Based Digitization of Protein-Self-Assembly to Print Biodegradable Physically Unclonable Labels for Device Security

**DOI:** 10.3390/mi14091678

**Published:** 2023-08-28

**Authors:** Sayantan Pradhan, Abhi D. Rajagopala, Emma Meno, Stephen Adams, Carl R. Elks, Peter A. Beling, Vamsi K. Yadavalli

**Affiliations:** 1 Department of Chemical and Life Science Engineering, Virginia Commonwealth University, Richmond, VA 23284, USA; pradhans3@vcu.edu; 2 Department of Electrical and Computer Engineering, Virginia Commonwealth University, Richmond, VA 23284, USA; rajagopalaad@vcu.edu (A.D.R.); crelks@vcu.edu (C.R.E.); 3 Intelligent Systems Division, Virginia Tech National Security Institute, Virginia Tech, Blacksburg, VA 24060, USA; emmam99@vt.edu (E.M.); scadams21@vt.edu (S.A.); beling@vt.edu (P.A.B.)

**Keywords:** physically unclonable function, device security, biodegradable label, deep learning, diffusion-limited aggregation, silk protein

## Abstract

The increasingly pervasive problem of counterfeiting affects both individuals and industry. In particular, public health and medical fields face threats to device authenticity and patient privacy, especially in the post-pandemic era. Physical unclonable functions (PUFs) present a modern solution using counterfeit-proof security labels to securely authenticate and identify physical objects. PUFs harness innately entropic information generators to create a unique fingerprint for an authentication protocol. This paper proposes a facile protein self-assembly process as an entropy generator for a unique biological PUF. The posited image digitization process applies a deep learning model to extract a feature vector from the self-assembly image. This is then binarized and debiased to produce a cryptographic key. The NIST SP 800-22 Statistical Test Suite was used to evaluate the randomness of the generated keys, which proved sufficiently stochastic. To facilitate deployment on physical objects, the PUF images were printed on flexible silk-fibroin-based biodegradable labels using functional protein bioinks. Images from the labels were captured using a cellphone camera and referenced against the source image for error rate comparison. The deep-learning-based biological PUF has potential as a low-cost, scalable, highly randomized strategy for anti-counterfeiting technology.

## 1. Introduction

The global problem of counterfeiting is one that causes significant financial damage and presents threats to individuals, companies, and governments. In particular, counterfeit products and biodevices that falsely represent their provenance are increasing in volume and sophistication [1]. Counterfeit and compromised medications, diagnostics, biodevices, or components susceptible to malfunction or malicious acts can harm both manufacturers and end-users. There is increasing urgency to ensure the implementation and improvement of high-security measures, e.g., securing supply chains, tracking or marking genuine devices, and having systems in place to trace and document suspected counterfeits [2,3]. Methods including holograms, barcodes, and quick response (QR) codes have been useful for various products [4,5]. For hardware components, manufacturing process variations have been proposed in the form of physically unclonable functions (PUFs) [6,7]. A PUF is a physical object with a unique, random intrinsic feature generated using a non-deterministic process [8]. This generates highly entropic information that can be used in a challenge-response pair mechanism, designed to be easy to evaluate but difficult to replicate. Thus, PUFs can act as fingerprints to certify the authenticity and reliability of products by presenting a unique security marker that can be associated with the object [9].

PUFs can be electrical or optical in form and provide secret fingerprints using manufacturing variations or stochastic processes [10,11]. PUFS exploiting silicon-integrated circuit variations (viz. Si-PUFs) have been proposed, but these are not suitable for non-electronic objects (e.g., medical devices, pharmaceuticals) [12]. To date, there has been limited work on addressing the issue of physical device/object security using easily printable PUF labels. Such labels can be used to verify authenticity and/or attribution by being affixed to a physical object. Optical PUFs in particular provide an interesting solution because they generate printable fingerprints based on visual inspection and image processing. This makes them beneficial for minimizing costs and providing broader access for end-user customer validation. However, optical PUFs require a physical entropic process as a starting point, for which various strategies have been proposed. For instance, the innate randomness in the spatial distribution of T-cell colonies (e.g., lymphocytes, or white blood cells) has been reported as a biological PUF [13]. Here, the PUFs utilize randomness in the spatiotemporal and collective behavioral dynamics of a biological species as the entropy generator [13]. An edible protein-based PUF was formed from fluorescent silk microparticles randomly scattered in a defined area. The unique set of excitation and emission bands of various fluorescent proteins is used as the input challenge, and images of spontaneous fluorescence are used as the response [14]. Such PUFs could be directly printed on pharmaceuticals as an anti-counterfeiting measure. Other biological PUF examples include randomly wrinkling silica-coated polymeric particles [15], randomly distributing Au nanodisks formed by nano-fracture using electron-beam lithography [16], and far-field scattering of randomly deposited Au nanoparticles [17]. A lens-free optical PUF utilizing stochastically manifested diffraction and native silk fibers has also been developed [18]. A biocompatible and flexible optical PUF label was developed by randomly embedding microdiamonds into silk fibroin films [19]. Notably, most methods reported in the literature require sophisticated materials or tools, such as fluorescence or high-resolution microscopes, for implementing authentication. The design and placement of image PUFs on physical objects using a relatively simple and low-cost strategy is still unaddressed. 

To address this challenge, we suggest that the process of protein self-assembly may be used as a readily available and facile entropy generator to form secure-by-design PUFs. As a proof-of-principle, we demonstrate a biological PUF built from the randomness of protein self-assembly. A high-resolution image of the self-assembly is printed on mechanically flexible labels. These labels can be physically affixed to a product and read using a cellphone camera. While being physically similar to holograms, barcodes, or QR codes, the proposed PUF labels have the added security of being extremely difficult to replicate (physically unclonable). In this demonstration, the labels are biodegradable over time, allowing for an added layer of tamper-evident, temporal protection. 

**Figure 1:**
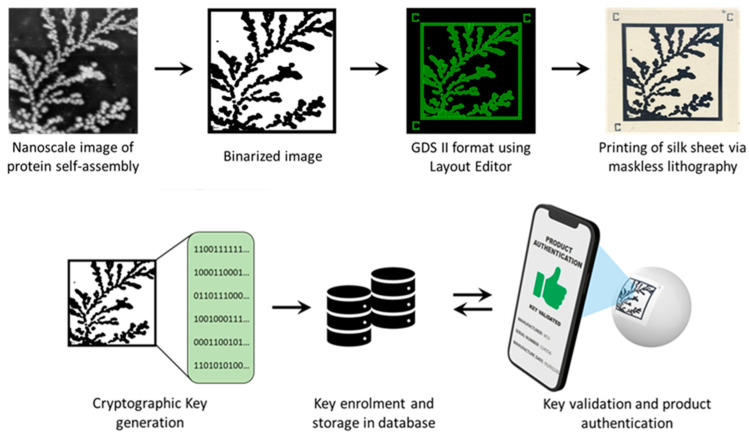
Schematic showing the proposed process for image PUF generation and printing.

Spontaneous organization across length scales to form larger, functional complexes via self-assembly is a phenomenon that is ubiquitous in nature [20]. Multiple weak, noncovalent interactions mediate this process, permitting the assembly of supramolecular structures from smaller building blocks [21]. Models have demonstrated that structures formed in this manner have a characteristic fractal dimensionality that is smaller than the embedding space [22,23]. In prior work from our group, we demonstrated how a protein named sericin, critical to silk macrostructure, displays the remarkable ability to self-assemble through different modes of classical and non-universal diffusion-limited aggregation (DLA) to produce radially branched dendritic architectures [24]. The autonomous assembly of this protein from an apparent lack of order into well-structured complexes can be used to create an entropic process. In this manuscript, we demonstrate the ability to generate counterfeit-proof security labels created using physical embodiments of this entropic process (Figure 1). Atomic Force Microscopy (AFM) was used to obtain high-resolution (nanoscale) images of the DLA protein assembly. The binarized images are fed into a deep learning network to extract a continuous feature vector corresponding to each image. After undergoing a digitization process involving binary quantization and randomness extraction, the result is a cryptographic key suitable for an authentication algorithm implementation. The original DLA (protein self-assembly) images can be directly printed on a biodegradable, mechanically flexible substrate using a functional bioink [25]. This allows the label to be affixed to a non-planar surface and read using a cellphone camera. The obtained image can then be compared to the cryptographic key for rapid verification and product identification. 

Our specific contributions to the field include: (1) a generalized method to generate keys from protein self-assembly via DLA; (2) an evaluation of using pre-trained deep learning models as a component of the process of converting the PUF image to a key; and (3) a maskless printing technique that prints the image on a biocompatible and biodegradable label. These easy-to-use security labels can therefore act as versatile digital signatures for the authentication of a wide variety of objects. This paper contributes to the fields of anti-counterfeiting, authentication, and deep learning by utilizing naturally occurring protein self-assembly as an entropy generator for a PUF. Using the principles shown in this work, any fractal assembly process may be used to form designs that are easily produced and evaluated and potentially provide a defense against duplication.

## 2. Materials and Methods

### 2.1. Materials

Sericin protein was obtained from the cocoons of *Bombyx mori* and is a commercially available product (Wako Fujifilm, Richmond, VA, USA). The fractal assembly of the silk protein sericin was used to generate the patterns used for the PUFs [24]. First, the protein powder was dissolved in molecular biology-grade deionized water to create a 2 mg/mL stock solution. The solution was vortexed at low speed for 30 min to ensure complete dissolution. Finally, 0.20 µm nylon syringe filters were used to remove large insoluble particulates, and the resulting solutions were stored at 2 °C. Hydroxylated p-type silicon (111) substrates were used to observe the protein self-assembly. Si substrates were treated with a mixture of 3:1 98% H_2_SO_4_:30% H_2_O_2_ to remove organic contaminants and render the substrate hydrophilic. The surface was washed with deionized water and ethanol and then dried at 150 °C. In the final step, 5 µL of solubilized protein was placed on the clean Si substrates and allowed to air-dry overnight at room temperature.

### 2.2. Fractal Pattern Acquisition via Multiscale Imaging

The proteins assemble on the surface via the process of diffusion-limited aggregation, forming unique fractal patterns [26,27]. Using a Nikon Eclipse LV100 optical microscope (Nikon, Japan), optical imaging of the air-dried samples was initially utilized to determine the fractal pattern of self-assembly in each sample. Samples were also observed using bright field microscopy with LU Plan Fluor lenses at 10× and 50× magnifications. Nanoscale images of the fractal self-assembly were obtained using AFM on an Asylum Research MFP-3D AFM (Asylum Research, Santa Barbara, CA). Imaging was performed in non-contact mode using an AC240TS AFM probe (Olympus, Japan) (nominal spring constant k ~2 nN/nm). The tip was operated at an optimal driving frequency of ~73 kHz and a scan rate of 0.80 Hz. The images were collected over a range of scan sizes (1–90 µm), and the minimum resolution was 512 × 512 points. The AFM images were obtained for the purpose of observing highly detailed fractal assemblies. As a ubiquitous natural process, *any* high-resolution images of self-assembly formed by DLA can be used. These may be taken by optical microscopy or even high-resolution cameras. The images are binarized for printing secure PUF labels, as discussed below.

### 2.3. Fabrication of Flexible and Biodegradable PUF Labels

The PS-PEDOT: PSS ink is spin-coated on the PF substrate formed on a Si wafer. The ink is dried on a hot plate at 65 °C for 5 min. Binarized images were then printed using Heidelberg instruments µMLA, a direct-write (maskless) photolithography tool. Exposure is carried out using a 365 nm LED @ 1500 mJ/cm^2^. Samples were subjected to a post-exposure baking (PEB) step at 110 °C for 1 minute to complete the photoreaction that takes place during the exposure. The patterns were developed using deionized water and removed/delaminated from the Si wafer to form a final free-standing, flexible PUF label.

## 3. Results

### 3.1. Printed Image PUFs

The image PUF used in this work is based on the 2D DLA of a protein on a flat surface [28]. In this demonstration, the protein sericin from Bombyx mori is used to replicate the phenomenon [29]. The DLA concept models ubiquitous natural processes by which matter irreversibly combines to form structures such as dendrites [30]. In prior work, drying-induced self-assembly of an aqueous solution of silk sericin into radially branched, dendritic architectures was observed on silicon substrates [24]. Colloidal particles diffuse into a growing cluster and act as the rate-limiting step of aggregate formation. The outward-branching aggregation via irreversible coalescence of colloidal particles is best described using a classical and non-universal DLA model, resulting in entropic radially-branched dendritic architectures with a fractal dimension [24]. This fractal architecture of branched structures is similar to a snowflake. Each sample is perfectly unique and therefore ideally suited to create an entropic process to enable physical data security. High-resolution fractal images of 2D sericin self-assembly were used as the source for the PUF labels. In this work, high-resolution images were obtained using AFM. It is important to note that DLA is very common in nature and often visible using regular microscopic tools (a high-resolution camera or even the naked eye) [31]. Thus, our presented strategy is widely applicable without the use of specialized tools. 

Prior to printing the labels on the substrate, the images of fractal self-assembly were processed (Figure 1). First, images were binarized using Otsu Processing [32]. The binarized images were converted to a graphical data system (GDS II) file format using Layout Editor. GDS II is a database file format that is the de facto industry standard for the data exchange of integrated circuits (IC) or IC layout artwork. The data was used to form the design of the printed label. Fiducial markers, such as “+” or “C”, were placed at three corners (top left, top right, and bottom left) of the “binarized images” in the GDS II format. This aids in pattern recognition, orientation, and stabilization of the field of view. Following these three steps, any image can be easily converted to a printable file format compatible with standard lithography tools. 

The printable files are then transformed into biofriendly PUF labels using a high-resolution microfabrication technique called silk protein photolithography (SPL). Our lab has previously demonstrated rapid SPL using photocrosslinkable bioinks [25,33,34,35,36,37]. The PUF labels are formed using a biodegradable dark protein bioink (image) on a flexible protein substrate. Details of the composition of these two elements of the label are provided in Section 2 [25]. The dark blue color of the PEDOT:PSS bioink provides a visible contrast for easy readability of the PUF labels using a standard cellphone camera. Although the conductive property of the PEDOT:PSS is inconsequential in this work, it paves the way for next-generation applications wherein the PUF labels can act as biodevices themselves. Notably, the ink itself can degrade in a proteolytic environment. The PUF labels are mechanically robust and can be handled with ease. They are optically transparent, flexible, and can conform even to non-planar, curvilinear surfaces (Figure 1). This allows for the PUF security label to be easily attached to a wide range of objects, such as hardware, medical devices, pharmaceuticals, and perishables. The scalability of the lithography process to print labels at different scales increases the target application repertoire. 

### 3.2. Cryptographic Keys

**Figure 2:**
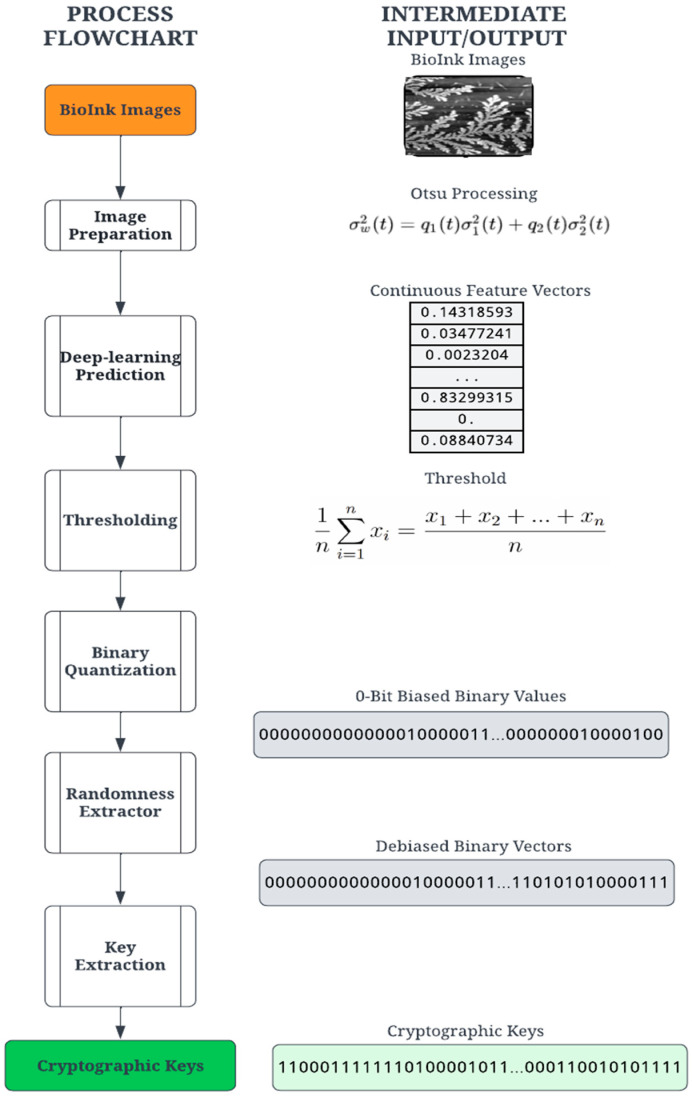
Cryptographic key generation process to create PUFs from the image of self-assembly.

The proposed digitization process transforms the PUF into secure binary digits for cryptographic computation. The process consists of a series of transformation stages on the entropy source, as shown in Figure 2. The first step applies common image processing techniques to the self-assembly image. The image is then passed through a deep-learning model (deep neural network), which converts the image into a vector of floating-point numbers. Finally, the digitization process converts these floating-point numbers into binary digits through a sequential process of thresholding, quantification, extraction, and key derivation. For this demonstration, a set of 54 PUF devices (using 54 self-assembly images) was created using the methods discussed below. 

#### 3.2.1. Image Processing

During the image processing stage, the PUF image is binarized using Otsu’s thresholding concept [32]. A variety of image thresholding techniques are available and were tested for this application. The first is global thresholding, where threshold values are dependent on the selected image. The primary drawback was selecting the threshold value per image, which introduced some non-determinism. Another technique, adaptive thresholding, selects an arbitrary frame size to calculate a mean within the embeddings. This process is also image-dependent but is a suitable alternative to global thresholding. Otsu’s method utilizes an image histogram to extract threshold values while also including filters to reduce noise [32]. During evaluation, Otsu’s method produced the clearest PUF images and was determined to be the most feasible for printing the PUF. Each PUF image undergoes image processing before being passed to the deep learning model.

#### 3.2.2. Deep Learning

The proposed solution incorporates deep learning via the deep neural network to convert the preprocessed PUF image into a vector of floating-point numbers. Recently, deep learning and cryptography have become increasingly connected, particularly in the face of quantum computing [38]. Traditionally, one-way mathematical functions (i.e., hashes) are robust and random enough to withstand brute-force decryption attacks and subsequent compromise. Harnessing deep learning models in the proposed solution introduces additional configurability and randomness in the PUF digitization process. Deep learning models are complex architectures composed of multiple layers, connections, and nodes. Such an architecture supports the ability to add layers, change sizes, and edit nodes. There are readily available architectures as well as more configurable options, increasing randomness and the ability to readjust in the face of a security breach. 

**Figure 3:**
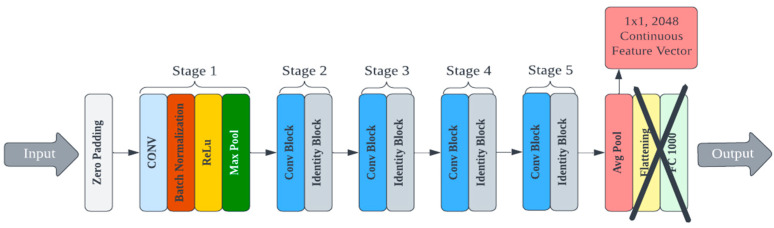
An example of an edited architecture for the ResNet50 model.

A custom deep learning model for feature extraction could be trained and used for cryptographic key generation. However, this process would require a large training dataset and significant computational resources devoted to architecture design and hyperparameter tuning. There are multiple pre-trained convolutional neural networks (CNNs) publicly available that have demonstrated superior performance for image processing [39,40]. Other deep hashing approaches (i.e., using deep learning for image hashing) require training at some step of the algorithm [41]. We use a lightweight deep hashing technique since much of the PUF’s randomness is derived from the protein’s self-assembly itself. Eight pre-trained models were selected from the Keras library for testing and evaluated on their ability to generate secure PUFs from bioInk images [42]. Each model differs in its efficiency and footprint, and these factors were also considered because the end goal is deployment to a mobile device. The architecture of these pre-trained models had to be reconfigured to output the feature vector values instead of the classification label. This was achieved by removing the linear layers associated with classification at the end of the network (the last five layers from MobileNet and the last two layers of all other models). An example of an edited architecture for the ResNet50 model is shown in Figure 3. The deep learning models were applied to the AFM image library to generate cryptographic keys. This process was repeated for each of the eight selected Keras models. Complete details on each model, the specifications, and the corresponding data are presented in the supporting information. 

Since the goal of the digitization process is a binary cryptographic key, it is necessary to derive a binary set of digits. The feature vector created by the deep learning model contains floating-point elements and must be converted to arrays of 0- and 1-bits. The quantization requires a threshold value for comparison, an average value based on the image. Given a feature vector $X=\left\{\;x_{1},\;x_{2},\ldots ,x_{n}\right\}$, the threshold ϵ is calculated using the mean of the feature values defined in Equation (1). It should be noted that the length n of the feature vectors is dependent on the network architecture.

(1) $$\epsilon =\frac{1}{n}\sum_{i=1}^{n}x_{i}$$

The quantization stage converts the floating-point vector into a binary vector by comparing the individual vector values with the threshold value ϵ; a value higher than the threshold results in 1, and a value lower than or equal to the threshold is a 0. Thus, binary quantization converts floating-point vector values into a sequence of n-bit binary numbers. Due to the nature of the image structure and feature vector calculation, the binary vectors were found to be significantly biased towards 0 bits and thus not sufficiently random for a cryptographic key. To ensure more statistical randomness and thus increased security of the cryptographic key, a classic Von Neumann extractor step is performed on the binary vector, producing a random and unbiased “strong key” [43]. 

The Von Neumann extractor yields a binary number by comparing two consecutive digits. For instance, the pair [0, 1] results in 1, the pair [1, 0] results in 0, and the pairs [0, 0] and [1, 1] are ignored. This process creates a debiased binary vector with a variable length shorter than the input vector. A cryptographic key suitable for an authentication algorithm was constructed by extracting the leading bits from the debiased binary vector. For this experiment, the key lengths tested were 256 and 128 bits. Given the feature vector lengths and Von Neumann debiasing algorithm, some images did not yield binary vectors long enough to satisfy the key length requirement. The extractor generates a variable-length key depending on the probability of consecutive pairs. A variable length is unusable for most cryptographic functions and requires a 64, 128, or 256-bit key length. A key derivation function (KDF) was implemented to create a constant key size using the result of the extractor. The KDF computes the size by comparing the size of each key in the set. For example, the minimum size for the tested sample set was 332 bits. The minimum size determines the largest possible key size, 256 bits, and truncates the rest of the bits from each key in the set. This process yields a 256-bit identity for each bioink image, which is random, unbiased, and suitable for cryptographic use. Our goal in this paper is to outline a ready-to-use methodology based on a starting entropic source image. We note that the steps outlined can be obtained rapidly using automated protocols and can be easily extended for larger cryptographic keys as needed. 

### 3.3. Performance of the PUF Devices

**Figure 4:**
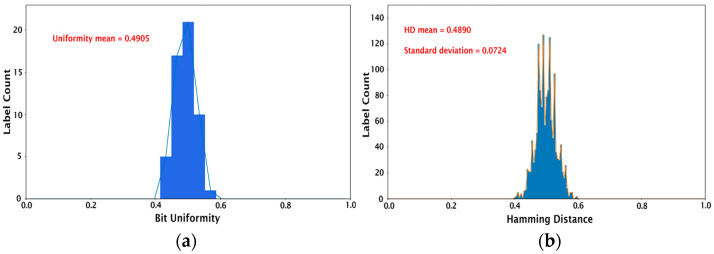
Histograms showing (**a**) bit uniformity calculation and (**b**) inter-hamming distance for the set of 54 PUFs studied.

Once the keys (256-bit cryptographic keys for 54 different PUFs) were created as described in the previous section, their performance was assessed using the following tests: (1) calculating device uniqueness and bit uniformity, and (2) validation using the NIST statistical test suite [44]. We note that these evaluation protocols for the image PUFs are standard in the field and have been used in prior works in the literature [13,14,15,16,17,18,19]. The bit uniformity, also known as Hamming weight, refers to the balance between the 1-bit and 0-bit states in the key. For example, a uniform key will have an equal number of zeros and ones. The bit uniformity calculation involves summing all the bits in the key and dividing by the number of bits as follows:

(2) $$Bit\;Uniformity=\frac{1}{n}\;\sum_{i=1}^{n}P_{i},$$

where $P_{i}$ is the $i^{th}$ binary bit, and n is the length of the key. The ideal bit uniformity value is 0.5. The bit uniformity calculation for our set of 54 PUF devices results in a mean value of 0.4905 and a median of 0.5, as shown in Figure 4a. The device uniqueness compares the 54 PUF devices with each other by calculating the Hamming distance (HD). Here, inter-HD is used since it describes the number of different bits between two PUFs. For a strong key, there should be no correlation between two PUFs, i.e., it should be impossible to predict the key from a PUF device even if the digitized keys from all the other PUFs are known. The inter-HD is given by:

(3) $$Inter\;HD=\frac{2}{m(m-1)}\;\sum_{j=1}^{m-1}\sum_{k=1}^{m}\frac{HD\;(P_{j},\;P_{k})}{n},$$

where $P_{j}$ and $P_{k}$ are the *n*-bit keys of the PUFs j and k, and m is the number of PUF devices. The comparison of 54 PUF devices generates 1431 unique HDs (^54^C_2_). The histograms in Figure 4b represent the HDs for each image sample. The mean HD for 1431 HDs is 0.4890, with a standard deviation of 0.0724, which is close to the ideal inter-HD of 0.5. This demonstrates the feasibility of producing PUF devices with excellent uniqueness using the proposed protein self-assembly as an entropy generation process.

To further evaluate the cryptographic strength of the PUFs, the NIST Statistical Test Suite was employed [44]. NIST SP 800-22 outlines a series of statistical tests for measuring the suitability of random and pseudorandom number generators for cryptographic applications. These tests are presented as a first step to determining feasibility in cryptography, noting that statistical testing is not a substitution for cryptanalysis [44]. For this study, six random number generation tests were selected for the evaluation of the binary cryptographic key strength. These tests (frequency test, frequency test within a block, cumulative sums test, runs test, test for the longest run of ones, approximate entropy test) have been previously reported in the validation and evaluation of keys for various forms of optical PUFs. Details are provided in the supporting information. 

Each of these statistical tests was implemented on the 256- and 128-bit cryptographic keys generated by the steps above. For each of the pre-trained deep learning models, the proportion of binary sequences passing the given statistical test is shown in Table S2 (which converts proportions into percentages). Table S2 displays the proportion of binary sequences passing the selected NIST statistical test for each of the key lengths and deep learning architectures tested. The “null” entries indicate that a model failed to generate a long enough cryptographic key for all images in the library. Details on the randomness tests per NIST SP 800-22 are provided in the supporting information [44]. The only deep learning model failing to pass a statistical proportion benchmark (exempting those failing to meet the key length requirement) was the 128-bit ResNet50 for the approximate entropy test (Table S2). The remainder of the models performed satisfactorily, showing the validity of this entropic generator. The *p*-value for the statistical test was used as a measure of the strength of evidence against the null hypothesis under test, i.e., the test sequence is random. Thus, the *p*-value represents a quantitative measure of the key’s randomness, where a *p*-value of 1 indicates a perfectly random key and a *p*-value of 0 indicates a completely non-random key. Table S3 summarizes the *p*-value distribution results of the statistical randomness tests used to analyze uniformity.

The deep learning model yielding the cryptographic key with the highest average *p*-value and thus highly random behavior was InceptionV3 (256-bits). Further, InceptionV3 had the smallest size in MB and one of the smallest times per inference step in both CPU and GPU, as shown in Table S1, priming it for lightweight implementations. ResNet50, one of the most widely used architectures in computer vision, also produced a set of cryptographic keys with a high average *p*-value (Table 1). Conversely, it has the largest size in MB and one of the longest times per inference step in CPU and GPU. When analyzing which models are best suited for cryptographic applications of image PUFs, it is important to consider the key length as a limiting factor. For example, both the Xception and DenseNet121 models generate 128-bit cryptographic key samples with high average *p*-values, but there may be a security tradeoff in weighing a 128-bit key vs. a 256-bit key. The relative importance of size specifications and cryptographic security must be considered and weighed by the implementer. Note further that a larger sample size is also recommended before selecting a single deep learning model for the PUF generation algorithm.

### 3.4. Key Authentication

**Figure 5:**
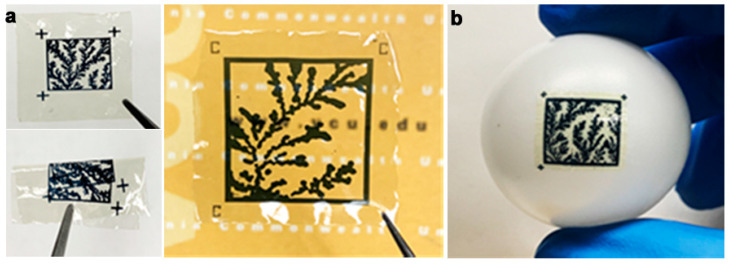
(**a**) The PUFs printed on flexible biodegradable substrates and can be rolled or folded. Fiduciary markers shown on the PUF labels. (**b**) Label directly affixed to a non-planar surface.

Authentication is the final step, where the user verifies the authenticity of a product via the PUF. One of the goals of facilitating the deployment of image PUFs is to simplify the process without requiring sophisticated or expensive tools. In prior reports, PUFs could be generated but were not easily affixed to physical objects [13,14,15,16,17,18]. In this work, we accomplish this by printing the binarized images using a bioink on a flexible, biodegradable substrate. The substrate is an optically transparent thin protein film, and the dark printable bioink provides a high-contrast label that can be attached even to non-planar objects (Figure 5). The validation process starts with capturing the bioink image on a cellphone. The image validation process compares this image with the existing image database constructed during the verification stage. The first step is image preparation, where OpenCV functions transform the image into the appropriate size, color, and other image properties required for binarization. For the current iteration, the pre-processed image runs through the same deep learning model as the database image to generate cryptographic keys. The image captured from a cellphone may have variable conditions such as camera resolution, lighting, etc., making complete binary matching difficult. Therefore, it is important to create useful threshold criteria to authenticate the images based on observation. The use of geometric distortion-invariant image hashing schemes can also provide for better authentication of digital images [45].

### 3.5. Validation

The above protocol was tested with four printed bioink PUF labels. An iPhone camera (iPhone 10, Apple) was used to capture two sample images for each label. The iPhone image follows the binarization process with the ResNet50 model to generate a binary key. The image validation conducts a bitwise comparison of the iPhone image and the database image via the binary keys. Table 2 shows the degree of matching (100% is a perfect match) for each PUF tested. In the figure, the original source image and the printed PUF are shown together with the degree of matching of each image taken with the cellphone camera. The fiduciary markers are used to reconcile any arbitrary orientation of the image and compare it to the source image. This process produced excellent results with an error rate of <10% (a match of 90.6 ± 1.8%). Given that this is a proof-of-principle work, we believe that these results can be further improved using other image processing and printing techniques in the future. 

## 4. Discussion

We have reported on using a ubiquitous protein self-assembly process (DLA) as a facile yet sophisticated entropy generator. The DLA provides source images for a printable PUF. In this work, imaging was conducted at the nano- and microscales using an AFM. However, in theory, it is possible to utilize imaging via an optical microscope or even a camera to obtain macroscale images. Since the self-assembly process is truly entropic, it is possible to obtain a unique image each time. Next, using a novel protein-based bioink, we demonstrate how the image can be printed on a mechanically flexible label using maskless lithography. The use of a biodegradable silk protein provides a sustainable and bio-friendly approach to physical data security. The silk labels themselves are easily attached to a variety of surface shapes and object types. The optical contrast provided by the dark bioink on the transparent PUF label allows for imaging using a cellphone camera. Notably, the bioink itself is biodegradable as well. Previously, silk-based biocompatible and flexible optical PUF labels were shown. For instance, by randomly embedding microdiamonds or by the random distribution of fluorescent particles [14,19]. We note that in both of these cases, specialized tools are required to read the films. More interestingly, label generation, key generation, and validation all have to be performed in the same setting. In this case, since the images are generated by self-assembly and then printed on the label, it is possible to separate the label and key design from the fabrication step, thereby providing a more decentralized operational modality. 

A deep-learning-based digitization protocol was used to verify the PUF’s suitability. The digitization algorithm generates a cryptographic key from the image using deep-learning prediction and a randomness extractor. The proposed digitization process produces sufficiently random and secure binary keys across a variety of deep-learning model architectures. Bitwise comparisons of the 2048-bit biased binary vectors produced by the original self-assembly image and a cellphone camera capture yield an error rate of <10%. This number has to be improved before such a system can be reliably deployed in the field. We note that the purpose of this work is to primarily demonstrate the feasibility of this technique for generating optical PUFs. It is the first step towards fabricating such easy-to-use, secure-by-design systems. In future work, larger sample sizes and cryptographic keys could be determined. This can further bring down the error rate to a very low number. Additionally, further analysis can be done to extrapolate an error function and ensure equivalency between the printed and digital images. This may involve testing a protocol based solely on the printed PUF labels. The use of geometric distortion-invariant image hashing schemes can be employed for better authentication of digital images [45]. Our ultimate goal is to develop a full authentication protocol using the PUF key implemented in a software application, allowing a user-facing avenue for counterfeiting protection.

We have shown that a low-cost rapid printing and image validation strategy can be used in conjunction with this entropic generator to form a system that is deployable in the real world. It is easy to envision a set of DLA images forming unique identifiers for product traceability, which can be tracked and compared once read by the user. Once used, they would be removed from the database, thereby allowing only *one-time* use of each PUF label. An additional feature of the flexible protein substrates (with the printed protein bioink) is their inherent biocompatibility and biodegradability. Thus, the PUF labels can themselves be programmed to degrade over time, which allows for an added layer of tamper-evident and aging protection. The labels can also be attached to a range of devices, including biomedical devices and perishables.

## 5. Conclusions

In summary, the development of secure and easily printable physical unclonable functions (PUFs) using protein self-assembly as an entropy generator offers a promising solution for ensuring product authentication. We have demonstrated the feasibility of using protein self-assembly, specifically the fractal assembly of the silk protein sericin, to generate unique PUF labels. High-resolution images of protein self-assembly were obtained using atomic force microscopy (AFM) and processed to create printable designs. The digitization process involved image processing and deep learning techniques to convert the images into cryptographic keys suitable for authentication algorithms. The generated PUF labels were printed on biodegradable and flexible substrates, allowing for easy attachment to various objects. The performance evaluation of the PUFs showed excellent uniqueness and bit uniformity, indicating their suitability for cryptographic applications. Additionally, the NIST Statistical Test Suite confirmed the randomness and cryptographic strength of the generated keys. The validation process using cellphone camera images demonstrated a low error rate and the potential for real-world deployment. Overall, this research contributes to the field of anti-counterfeiting and authentication by introducing a novel approach that combines protein self-assembly, deep learning, and bioink printing to create secure and easily deployable PUF labels for a wide range of high-value objects.

## Figures and Tables

**Table 1:** NIST Statistical Test Suite *p*-Value distribution results for ResNet50 for the 54 PUFs.

NIST Statistical Test	*p*-Value	Proportion	Result
Frequency	0.040108	54/54	Pass
Block Frequency	0.574903	54/54	Pass
Cumulative Sums	0.000274	54/54	Pass
	0.023545	54/54	Pass
Runs	0.137282	54/54	Pass
Longest Run of Ones	0.883171	54/54	Pass
Approximate Entropy	0.574903	53/54	Pass

**Table 2:** Error Rate comparison between AFM and printed PUF label images.

Sample #	AFM Image	Printed Label	% Match with AFM Image	
			Image #1	Image #2
1	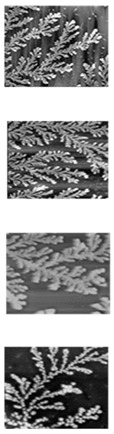	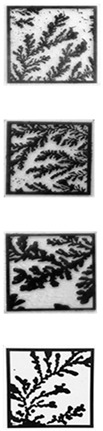	90.7%	89.8%
2			89.4%	89.5%
3			93.3%	93.7%
4			89.2%	89.3%

## Data Availability

Data is available from the authors by request.

## Supplementary Materials

The following supporting information can be downloaded at: https://www.mdpi.com/article/10.3390/mi14091678/s1. Deep Learning Algorithm, Key Validation Tables, and Binary Quantization Algorithm. Table S1: Keras Model Performance Statistics and Specifications; Table S2: NIST Statistical Test Suite Proportion Results (in green is the selected model); Table S3: NIST Statistical Test Suite *p*-Value Distribution Results (in green is the selected model). Ref [46] is cited in Supplementary Materials file.

## References

[1] Mackey T.K., Nayyar G. (2017). A review of existing and emerging digital technologies to combat the global trade in fake medicines. Expert Opin. Drug Saf..

[2] Zafar S., Nazir M., Bakhshi T., Khattak H.A., Khan S., Bilal M., Choo K.-K.R., Kwak K.-S., Sabah A. (2021). A systematic review of bio-cyber interface technologies and security issues for internet of bio-nano things. IEEE Access.

[3] Lewis T.G. (2019). Critical Infrastructure Protection in Homeland Security: Defending a Networked Nation.

[4] Soon J.M., Manning L. (2019). Developing anti-counterfeiting measures: The role of smart packaging. Food Res. Int..

[5] Picard J., Landry P., Bolay M. (2021). Counterfeit Detection with QR Codes. Proceedings of the 21st ACM Symposium on Document Engineering.

[6] Mukhopadhyay D. (2016). PUFs as promising tools for security in internet of things. IEEE Des. Test.

[7] Halak B., Zwolinski M., Mispan M.S. (2016). Overview of PUF-Based Hardware Security Solutions for the Internet of Things. Proceedings of the 2016 IEEE 59th International Midwest Symposium on Circuits and Systems (MWSCAS).

[8] Pappu R., Recht B., Taylor J., Gershenfeld N. (2002). Physical one-way functions. Science.

[9] Arppe R., Sørensen T.J. (2017). Physical unclonable functions generated through chemical methods for anti-counterfeiting. Nat. Rev. Chem..

[10] Erozan A.T., Hefenbrock M., Beigl M., Aghassi-Hagmann J., Tahoori M.B. (2019). Image PUF: A Physical Unclonable Function for Printed Electronics based on Optical Variation of Printed Inks. IACR Cryptol. Eprint Arch..

[11] Wigger B., Meissner T., Förste A., Jetter V., Zimmermann A. (2018). Using unique surface patterns of injection moulded plastic components as an image based Physical Unclonable Function for secure component identification. Sci. Rep..

[12] Zerrouki F., Ouchani S., Bouarfa H. (2022). A survey on silicon PUFs. J. Syst. Archit..

[13] Wali A., Dodda A., Wu Y., Pannone A., Reddy Usthili L.K., Ozdemir S.K., Ozbolat I.T., Das S. (2019). Biological physically unclonable function. Commun. Phys..

[14] Leem J.W., Kim M.S., Choi S.H., Kim S.-R., Kim S.-W., Song Y.M., Young R.J., Kim Y.L. (2020). Edible unclonable functions. Nat. Commun..

[15] Bae H.J., Bae S., Park C., Han S., Kim J., Kim L.N., Kim K., Song S.H., Park W., Kwon S. (2015). Biomimetic microfingerprints for anti-counterfeiting strategies. Adv. Mater..

[16] Zhang T., Shu Z., Zhang L., Chen Y., Feng Z., Hu Y., Huang F., Wang P., Li D., Yao Y. (2021). Random Nanofracture-Enabled Physical Unclonable Function. Adv. Mater. Technol..

[17] Smith A.F., Patton P., Skrabalak S.E. (2016). Plasmonic nanoparticles as a physically unclonable function for responsive anti-counterfeit nanofingerprints. Adv. Funct. Mater..

[18] Kim M.S., Lee G.J., Leem J.W., Choi S., Kim Y.L., Song Y.M. (2022). Revisiting silk: A lens-free optical physical unclonable function. Nat. Commun..

[19] Hu Y.W., Zhang T.P., Wang C.F., Liu K.K., Sun Y., Li L., Lv C.F., Liang Y.C., Jiao F.H., Zhao W.B. (2021). Flexible and biocompatible physical unclonable function anti-counterfeiting label. Adv. Funct. Mater..

[20] Whitesides G.M., Grzybowski B. (2002). Self-assembly at all scales. Science.

[21] Philp D., Stoddart J.F. (1996). Self-assembly in natural and unnatural systems. Angew. Chem.-Int. Ed. Engl..

[22] Bohr H., Kühle A., Sørensen A.H., Bohr J. (1997). Hierarchical organization in aggregates of protein molecules. Z. Für Phys. D At. Mol. Clust..

[23] Meakin P., Jullien R. (1988). The Effects of Restructuring on the Geometry of Clusters Formed by Diffusion-Limited, Ballistic, and Reaction-Limited Cluster Cluster Aggregation. J. Chem. Phys..

[24] Khire T.S., Kundu J., Kundu S.C., Yadavalli V.K. (2010). The fractal self-assembly of the silk protein sericin. Soft Matter.

[25] Pal R.K., Farghaly A.A., Collinson M.M., Kundu S.C., Yadavalli V.K. (2016). Photolithographic Micropatterning of Conducting Polymers on Flexible Silk Matrices. Adv. Mater.

[26] Tokuyama M., Kawasaki K. (1984). Fractal Dimensions For Diffusion-Limited Aggregation. Phys. Lett. A.

[27] Choi J., Crowdy D., Bazant M.Z. (2010). Diffusion-limited aggregation on curved surfaces. EPL (Europhys. Lett.).

[28] Hurd A.J., Schaefer D.W. (1985). Diffusion-limited aggregation in two dimensions. Phys. Rev. Lett..

[29] Kato N., Sato S., Yamanaka A., Yamada H., Fuwa N., Nomura M. (1998). Silk protein, sericin, inhibits lipid peroxidation and tyrosinase activity. Biosci. Biotechnol. Biochem..

[30] Witten Jr T.A., Sander L.M. (1981). Diffusion-limited aggregation, a kinetic critical phenomenon. Phys. Rev. Lett..

[31] Sander L.M. (2000). Diffusion-limited aggregation: A kinetic critical phenomenon?. Contemp. Phys..

[32] Goh T.Y., Basah S.N., Yazid H., Safar M.J.A., Saad F.S.A. (2018). Performance analysis of image thresholding: Otsu technique. Measurement.

[33] Kurland N.E., Dey T., Kundu S.C., Yadavalli V.K. (2013). Precise Patterning of Silk Microstructures Using Photolithography. Adv. Mater..

[34] Kurland N.E., Dey T., Wang C., Kundu S.C., Yadavalli V.K. (2014). Silk protein lithography as a route to fabricate sericin microarchitectures. Adv. Mater.

[35] Xu M., Yadavalli V.K. (2019). Flexible Biosensors for the Impedimetric Detection of Protein Targets Using Silk-Conductive Polymer Biocomposites. ACS Sens..

[36] Pal R.K., Farghaly A.A., Wang C., Collinson M.M., Kundu S.C., Yadavalli V.K. (2016). Conducting polymer-silk biocomposites for flexible and biodegradable electrochemical sensors. Biosens. Bioelectron..

[37] Pal R.K., Kundu S.C., Yadavalli V.K. (2017). Biosensing using photolithographically micropatterned electrodes of PEDOT:PSS on ITO substrates. Sens. Actuators B Chem..

[38] Alani M.M. (2019). Applications of machine learning in cryptography: A survey. Proceedings of the 3rd International Conference on Cryptography, Security and Privacy.

[39] Shen F., Xu Y., Liu L., Yang Y., Huang Z., Shen H.T. (2018). Unsupervised deep hashing with similarity-adaptive and discrete optimization. IEEE Trans. Pattern Anal. Mach. Intell..

[40] Liu N., Mou H., Tang J., Wan L., Li Q., Yuan Y. (2022). Fully Connected Hashing Neural Networks for Indexing Large-Scale Remote Sensing Images. Mathematics.

[41] Lin K., Yang H.-F., Hsiao J.-H., Chen C.-S. Deep learning of binary hash codes for fast image retrieval. Proceedings of the IEEE Conference on Computer Vision and Pattern Recognition Workshops.

[42] Team, Keras (2020). Keras Documentation: Keras Applications. https://keras.io/api/applications/.

[43] Seepers R.M., Strydis C., Sourdis I., De Zeeuw C.I. (2015). On using a von neumann extractor in heart-beat-based security. Proceedings of the 2015 IEEE Trustcom/BigDataSE/ISPA.

[44] Rukhin A., Soto J., Nechvatal J., Smid M., Barker E. (2001). A Statistical Test Suite for Random and Pseudorandom Number Generators for Cryptographic Applications.

[45] Lu C.-S., Hsu C.-Y. (2005). Geometric distortion-resilient image hashing scheme and its applications on copy detection and authentication. Multimed. Syst..

[46] Ketkar N. (2017). Introduction to keras. Deep Learning with Python: A Hands-On Introduction.

