# Supplementary material for: Deep-Learning-Based Digitization of Protein-Self-Assembly to Print Biodegradable Physically Unclonable Labels for Device Security
Source: Micromachines (Basel). 2023 Aug 28;14(9):1678. doi: 10.3390/mi14091678 (PMC10535045; doi:10.3390/mi14091678)
Supplement: Supplementary file 1 [file micromachines-14-01678-s001.zip › micromachines-2416645-supplementary.pdf]

Deep-learning-based digitization of protein-self-assembly to form biodegradable physically unclonable labels for device security

Sayantan Pradhan [a, *], Abhi D. Rajagopala [b, *], Emma Meno [c, *], Stephen Adams [c], Carl R. Elks [b], Peter A. Beling [c], Vamsi K. Yadavalli [a,#]

[*] - These authors contributed equally to the work

[a] Department of Chemical and Life Science Engineering, Virginia Commonwealth University

[b] Department of Electrical and Computer Engineering, Virginia Commonwealth University

[c] Intelligence Systems Division, Virginia Tech National Security Institute, Virginia Tech

[#] - Corresponding Author: vyadavalli@vcu.edu

Deep Learning Model

There are multiple deep learning models available for image processing. Each learning model differs in its efficiency and footprint. To analyze the relative security of different deep learning models in generating physically unclonable functions (PUFs) from the source images, eight models were selected from the Keras library for testing [1]. **Table S1**, content taken directly from the Keras library documentation page [2], details the model architecture information, performance statistics on a standard benchmark computer vision data set, and inference time on standard hardware. This information can be used to estimate the performance of a model on a deployed application.

**Model**	Feature Vector Length	Size (MB)	Top-1 Accuracy	Top-5 Accuracy	Params	Depth	Time (ms) per inference step (CPU)	Time (ms) per inference step (GPU)
ResNet50	2048	98	74.9%	92.1%	25.6M	107	58.2	4.6
ResNet50V2	2048	98	76.0%	93.0%	25.6M	103	45.6	4.4
MobileNet	1024	16	70.4%	89.5%	4.3M	55	22.6	3.4
InceptionV3	2048	14	71.3%	90.1%	3.5M	105	25.9	3.8
Xception	2048	88	79.0%	94.5%	22.9M	81	109.4	8.1
DenseNet121	1024	33	75.0%	92.3%	8.1M	242	77.1	5.4
DenseNet201	1920	80	77.3%	93.6%	20.2M	402	127.2	6.7
NASNetMobile	1056	23	74.4%	91.9%	5.3M	389	27.0	6.7
Average	----	56	74.7%	92.1%	14.4M	185	61.6	5.3

Table S1: *Keras Model Performance Statistics and Specifications [2]*

The size of the deep learning models are represented in megabytes (MB). The top-1 and top-5 accuracy statistics represent the select model's performance on the ImageNet validation dataset of 50000 images. The depth of the model represents the network's topological depth, counting the number of parameterized layers. The time per inference step was averaged over 30 batches and 10 repetitions with a 92 AMD EPYC Processor (with IBPB) CPU, 1.7T RAM, a Tesla A100 GPU,

and a batch size of 32 per the conditions. To compute the last row of the table, the arithmetic mean of each tested network's performance in the corresponding column is truncated to the appropriate decimal place. Note that the feature vector length is also included in Table 1, required to configure the key generation algorithm. As shown in **Table S1**, ResNet50, the first model tested on the PUFs dataset, measures the greatest size in megabytes and parameters, but the time per inference step (both CPU and GPU) is comparable to the average for this model dataset. Per the Keras API metrics, the most size efficient models tested were InceptionV3, MobileNet, and NASNetMobile and the most time efficient models were MobileNet, InceptionV3, and ResNet50V2.

The deep learning model is applied to the high-resolution images of protein self-assembly to generate cryptographic keys. This process was repeated for each of the eight select Keras models in a Python Jupyter notebook. The set of 54 images was converted to a data stream compatible for the deep learning models to ingest. Each image is individually loaded and plotted then converted into an array and reshaped. The image encodings are preprocessed to properly scale inputs for machine learning. The deep learning model was loaded and a feature list initialized. Note that the architecture of these predefined models had to be reconfigured to output the feature vector values, achieved by stripping the last five layers from MobileNet and last two layers of all other models. The model then iterates over each of the images to predict a feature vector, varying in length depending on the architecture, shown in the "Feature Vector Length" column in **Table S1**. The model prediction process outputs a continuous feature vector for each of the source images, where each element is a decimal value.

i) Thresholding: The first step in digitization is to find a threshold for binary quantization (0 and 1). The quantization requires a threshold value for comparison. Since the output of the learning model is a floating-point value, the thresholding finds an average value based on the image. The threshold value is the mean of the set of vector values. For each image, the value of ***n= 2048****,* since the ResNet-50 output has ***2048*** floating-point numbers (vector values). The threshold output will be a single floating-point number, the average of 2048 values. The thresholding removes any dependency of the source image with similar images; since the threshold value is on a particular image. This technique deviates from the other machine learning techniques, which compares different images for similarity. In this process, a simple average computation provides the

threshold. However, if the quality of the key is inadequate, as discussed in Section 3, then the thresholding requires complex computation.

ii) Quantization: The output of thresholding is a non-binary floating point number, but a cryptographic key is a binary number. The quantization stage converts the floating-point number into a binary number by comparing the individual vector values with the threshold value to generate the binary values. Algorithm 1 illustrates the quantization algorithm; a value higher than the threshold results in 1, and a value lower than or equal to the threshold is a 0. Since the vector has a 2048 floating-point value, the result is a 2048-bit binary number. Thus, binary quantization converts floating-pointing vector values into a sequence of binary numbers.

iii) Von Neumann Extractor for debiasing: The 2048-bit binary value from quantization is not random and is statistically biased, having an unfair number of zeros or ones. An extractor emanates a random and unbiased "strong key" for cryptography. The process uses a Von-Neumann extractor, which yields a binary number by comparing two consecutive digits [3]. As shown in Algorithm 2, the pair [0, 1] converts to 1, the pair [1, 0] results in 0, and [0, 0] and [1, 1] are ignored. The end result is a random debiased binary number suitable to generate a strong key.

iv) Key Derivation Function (KDF): The extractor generates a variable-length key depending on the consecutive pair probability. A variable length is unusable for most cryptographic functions and requires a 64, 128, or 256-bit key length. The KDF creates a constant key size using the result of the extractor. The KDF computes the size by comparing the size of each key in the set. For example, the minimum size for the tested sample was 332 bits. The minimum size determines the largest possible key size, 256 bits, and truncates the rest of the bits from each key in the set. This process yields a 256-bit identity for each source (self-assembly) image which is random, unbiased, and cryptographic.

Key Validation and Evaluation

The cryptographic strength of the binary keys generated from PUFs was evaluated using the NIST Statistical Test Suite [4]. NIST SP 800-22 outlines a series of statistical tests for measuring suitability of random and pseudorandom number generators for cryptographic applications. These tests are presented as a first step to determine feasibility in cryptography, noting that statistical testing is not a substitution for cryptanalysis [4]. For this experiment, six random number generation tests were selected for the evaluation of the binary cryptographic key strength:

1. *Frequency Test* measures whether the proportion of ones and zeros over the sequence is consistent with that expected from a truly random sequence, approximately 50% zeros and 50% ones.
2. *Frequency Test within a Block* measures the proportion of ones and zeros within a defined block length against an optimum 50% zeros and 50% ones. For this experiment, the block length is 128. Note then that for 128-bit keys, the frequency and block frequency tests produce the same result.
3. *Cumulative Sums Test* measures whether the cumulative sum of partial sequences is similar to the expected random sequence behavior, adjusting 0-bits to -1 and 1-bits to 1 and summing the maximum excursion of a random walk. This test produces an output in forward and reverse mode.
4. *Runs Test* measures the total number of uninterrupted sequences of identical bits in a sequence, bounded before and after a bit with the flipped value. This test utilizes a reference distribution to measure the oscillation between ones and zeros.
5. *Test for the Longest Run of Ones in a Block* measures whether the longest run of ones within blocks is comparable with the longest run of ones in blocks of a random sequence. Given the length of the bit strings is 128 and 256, the specification defines the length of each block as 8 bits.
6. *Approximate Entropy Test* measures the frequency of overlapping bit patterns measured across the whole sequence. The specification recommends the bit length be set such that $m < [log_2 n] - 5$ where *m* is the block length and *n* is the length of the entire bit sequence. Thus, for 256-bit keys, the test measures 2-bit patterns and for 128-bit keys, the test measures 1-bit patterns.

Table S2 shows the proportion of binary sequences passing the select NIST statistical test for each of the key lengths and deep learning architectures tested. The following confidence interval equation calculates the threshold of acceptable proportions of sequences passing the randomness tests: $\hat{p} \pm 3\sqrt{\frac{\hat{p}(1-\hat{p})}{m}}$, where $\hat{p}$= 1-α, and m is the sample size

**Model**	Key Length	Freq	Block Freq	Cumulative Sums		Runs	Longest Run	Approx Entropy
**ResNet50**	256-bit	100%	100%	100%	100%	98.15%	98.15%	100%
	128-bit	100%	100%	100%	100%	96.30%	100%	94.44%
ResNet50V2	256-bit	null	Null	null	null	null	null	null
	128-bit	100%	100%	100%	100%	98.15%	100%	100%
MobileNet	256-bit	null	Null	null	null	null	null	null
	128-bit	100%	100%	100%	100%	100%	100%	100%
InceptionV3	256-bit	100%	100%	100%	100%	98.15%	96.30%	100%
	128-bit	100%	100%	100%	100%	100%	100%	100%
Xception	256-bit	96.30%	98.15%	96.30%	96.30%	100%	98.15%	100%
	128-bit	100%	100%	100%	100%	100%	98.15%	100%
DenseNet121	256-bit	null	Null	null	null	null	null	null
	128-bit	100%	100%	100%	100%	100%	98.15%	100%
DenseNet201	256-bit	null	Null	null	null	null	null	null
	128-bit	100%	100%	100%	100%	98.15%	100%	100%
NASNetMobile	256-bit	null	Null	null	null	null	null	null
	128-bit	100%	100%	100%	100%	100%	98.15%	100%
Minimum Pass Rate	---------	94.94%	94.94%	94.94%	94.94%	94.94%	94.94%	94.94%

Table S2: *NIST Statistical Test Suite Proportion Results (in green is the selected model)*

Note that α is the significance level, i.e., the probability a conclusion rejects the null hypothesis. Per NIST SP 800-22 [2], the null hypothesis under test is that the test sequence behavior mimics

that of a random sequence, so α represents the probability the data is non-random. For this experiment, α is defined as 0.01 and $\hat{p}$ = 1-0.01 = 0.99. The sample size is 54 binary sequences. Given these experimental values, the minimum pass rate is:

$$0.99 - 3\sqrt{\frac{0.99(1-0.99)}{54}} = 0.9494 = 94.94\%$$

The proportion values failing to pass this benchmark are highlighted in red. From **Table S2**, the only deep learning model failing to pass a statistical proportion benchmark (exempting those failing to meet the key length requirement) was the 128-bit ResNet50 for the approximate entropy test.

**Model**	Key Length (bits)	Freq P-value$_r$	Block Freq P-value$_r$	Cumulative Sums P-value$_r$		Runs P-value$_r$	Longest Run P-value$_r$	Approx Entropy P-value$_r$	Avg P-value$_r$
ResNet50	256	0.040108	0.574903	0.000274	0.023545	0.137282	0.883171	0.574903	0.319169
	128	0.213309	0.213309	0.023545	0.011791	0.006661	0.657933	0.002758	0.161329
ResNet50 V2	128	0.020548	0.020548	0.011791	0.015598	0.455937	0.494392	0.010237	0.147007
MobileNet	128	0.035174	0.035174	0.319084	0.236810	0.262249	0.213309	0.213309	0.187873
Inception V3	256	0.883171	0.574903	0.000170	0.236810	0.534146	0.213309	0.319084	0.394513
	128	0.005762	0.005762	0.017912	0.023545	0.051942	0.816537	0.108791	0.147179
Xception	256	0.005762	0.005762	0.005762	0.005762	0.005762	0.005762	0.289667	0.046320
	128	0.262249	0.262249	0.262249	0.262249	0.262249	0.262249	0.657933	0.318775
DenseNet 121	128	0.085587	0.085587	0.035174	0.699313	0.419021	0.262249	0.657933	0.320695
DenseNet 201	128	0.015598	0.015598	0.007694	0.002043	0.040108	0.035174	0.494392	0.087230
NASNet Mobile	128	0.011791	0.011791	0.108791	0.096578	0.026948	0.350485	0.085587	0.098853

Table 3: *NIST Statistical Test Suite P-Value Distribution Results (in green is the selected model)*

Table S3 summarizes the P-value distribution results of the statistical randomness tests, used to analyze uniformity. The P-value represents the strength of evidence against the null hypothesis under test, i.e. the test sequence is random. Thus, the P-value represents a quantitative measure of the key randomness, where a P-value of 1 indicates a perfectly random key and a P-value of 0 indicates a completely non-random key. To determine if the distribution of the calculated P-values adheres to expected random behavior, a Goodness-of-Fit Distributional Test is run over the 54 calculated P-values to determine a "P-value of P-values" for the given statistical test. First, the distribution of P-values in intervals of 0.1 from 0 to 1 is calculated by applying a chi-squared test: $\chi^2 = \sum_{i=1}^{10} \frac{(F_i - s/10)^2}{s/10}$, where F_i is number of P-values in sub-interval *i* and *s* is sample size

For this experiment, the sample size *s* is 54, the number of cryptographic keys generated from the source images. The chi-squared value is then used to calculate the uniformity represented by the $P\text{-}value_r$ = **igamc**$(9/2, \chi^2/2)$ where **igamc** is the incomplete gamma function given by: $Q(a,x) = \frac{1}{\Gamma(a)} \int_x^{\infty} e^{-t} t^{a-1}\, dt$, and $\Gamma(a)$ is the gamma function given by: $\Gamma(a) = \int_0^{\infty} t^{a-1} e^{-t}\, dt$.

As cited by NIST SP 800-22 [2], if $P\text{-}value_r \geq 0.0001$, the sequences are considered uniformly distributed. Note from the specification that 55 sequences is considered the benchmark for statistical weight, but only 54 key samples were used for this experiment. This can be improved in future iterations.

Binary Quantization Algorithm

The binary quantization of the 54 one-dimensional continuous feature vectors was performed using the threshold as follows:

- If a feature value was greater than the threshold, that binary vector element became "1."
- If the feature value was less than or equal to the threshold, the binary vector element became "0."

Note that given the images and feature vector calculation, these binary vectors are significantly biased towards 0-bits.

Algorithm 1 Binary Quantization Algorithm

```
i ← 0
for value (i) in the vector do
    if i ≥ Threshold then
        binarydigit[i] ← 1
    else if i ≤ Threshold then
        binarydigit[i] ← 0
    end if
    i ← i + 1
end for
```

Von Neumann Extraction

This randomness extractor parses bit pairs of the input binary vector, performing the following for each:

- If the input is 00 or 11, these bits are discarded.
- If the input is 01 or 10, only the first bit is preserved (i.e., 0 for 01 and 1 for 10)

Algorithm 2 Extractor Algorithm to de-bias the number

```
j ← 0 i ← 0
for Binary value (i) in the vector do
    if value[i] is 0 and value[i + 1] is 1 then
        key[j] ← 1
        j ← j + 1
    else if value[i] is 1 and value[i + 1] is 0 then
        key[j] ← 0
        j ← j + 1
    end if
end for
```

References:

1. Ketkar, N. and N. Ketkar, *Introduction to keras.* Deep learning with python: a hands-on introduction, 2017: p. 97-111.
2. Team, Keras., *Keras documentation: Keras applications.* URL: https://keras.io/api/applications/, 2020.
3. Seepers, R.M., et al. *On using a von neumann extractor in heart-beat-based security*. in *2015 IEEE Trustcom/BigDataSE/ISPA*. 2015. IEEE.
4. Rukhin, A., et al., *A statistical test suite for random and pseudorandom number generators for cryptographic applications*. 2001, Booz-allen and hamilton inc mclean va.
